# A comparative study on decision and documentation of refraining from resuscitation in two medical home care units in Sweden

**DOI:** 10.1186/s12904-019-0472-z

**Published:** 2019-10-17

**Authors:** Bogdan Sterpu, Pia Lindman, Linda Björkhem-Bergman

**Affiliations:** 1 ASIH Stockholm Norr, Medical Home Care and Hospice Ward, Svärdvägen 11 D, SE-182 33 Danderyd, Sweden; 2 ASIH Stockholm Södra, Medical Home Care and Hospice Ward, Bergtallsvägen 12, SE-125 59 Älvsjö, Sweden; 3 Karolinska Institutet, Department of Neurobiology, Care Sciences and Society (NVS), Division of Clinical Geriatrics, Blickagången 16, Neo floor 7, SE-141 83 Huddinge, Sweden; 4 Karolinska Institutet and ASIH Stockholm Södra, Bergtallsvägen 12, SE-125 59 Älvsjö, Sweden

**Keywords:** Palliative care, Decision, Cardiopulmonary resuscitation, Documentation

## Abstract

**Background:**

A decision to refrain from cardiopulmonary resuscitation (CPR) in the case of cardiac arrest is recommended in terminally ill patients to avoid unnecessary suffering at time of death. The aim of this study was to describe the frequency of decisions and documentation of “do not attempt cardiopulmonary resuscitation” (DNACPR) in two Medical Home Care Units in Stockholm. Unit A had written guidelines about how to document CPR-decisions in the medical records, including a requirement for a decision to be taken (CPR: yes/no) while Unit B had no such requirement.

**Method:**

The medical records for all patients in palliative phase of their disease at the two Units were reviewed. Data was collected on documentation of decisions about CPR (yes/no), DNACPR-decisions and documentation regarding whether the patient or next-of-kin had been informed about the DNACPR-decision.

**Results:**

In the two Units, 316 and 219 patients in palliative phase were identified. In Unit A 100% of the patients had a CPR-decision (yes/no) compared to 79% in Unit B (*p* < 0.001). There was no statistically significant difference in DNACPR-decisions between the two Units, 43 and 37%. Documentation about informing the patient regarding the decision was significantly higher in Unit A, 53% compared to 14% at Unit B (*p* < 0.001). Documentation about informing the next-of-kin was also significantly higher at Unit A; 42% compared to 6% at Unit B (*p* < 0.001).

**Conclusion:**

Less than 50% of patients in palliative phase had a decision of DNACPR in two Medical Home Care Units in Stockholm. The presence of written guidelines and a requirement for a CPR-decision did not increase the frequency of DNACPR-decisions but was associated with a higher frequency of documentation of decisions and of information given to both the patients and the next-of-kin.

## Introduction

In Sweden, Medical Home Care Units offer hospital-like care at home to both oncological and non-oncological patients. Most of the enrolled patients suffer from a chronic, life-threatening disease, i.e. patients in palliative phase of a disease. A minority of the patients have other non-palliative conditions but need supportive medical care at home for some time. The overall aim of the Medical Home Care is to provide good palliative and/or supporting care to the patients in their own homes and to avoid hospital admissions. The Medical Home Care also facilitates the possibility to be cared for at home during the final days of life and allows patients to die at home. In this context the decision to refrain from cardiopulmonary resuscitation (CPR) in the case of cardiac arrest needs to be addressed and discussed with the patient and the next-of-kin [1, 2].

When CPR was first described in the 1960s as a life-saving action in people who experienced sudden cardiac arrest it was never intended to be used in terminally ill patients [3, 4]. However, it soon became a routine practice to perform CPR in all cases of cardiac arrest – also in patients with chronic illness, and even in dying patients [4]. Yet, the decision to refrain from CPR is often difficult to take for the physician and is an emotionally charged discussion to have with the patients and the next-of-kin [5]. Most importantly, CPR might result in unnecessary suffering for the dying patient [4].

The outcome of CPR is generally poor in most people and the survival rate varies between 10 and 20% according to studies from different countries around the world [2, 6–8]. However, the outcome of CPR is even worse in patients with cancer or other severe illnesses. In a systematic review including 31 studies it was shown that cancer patients had a survival rate of 6% following CPR, and the survival rate was as low as 2% in patients with advanced cancer [4]. In a review comprising 18 studies on the success rate of in-hospital CPR in patients with advanced cancer, being in palliative phase of the disease, i.e. the same kind of patients enrolled in Medical Home Care in Sweden, the success rate was close to zero [9]. However, it is rather common that a decision to “do not attempt cardiopulmonary resuscitation” (DNACPR) has not been taken been taken in this group of patients.

According to the Swedish law, it is advisable but not absolutely necessary that a DNACPR-decision is taken in agreement with the patient (http://www.riksdagen.se/sv/dokument-lagar/dokument/svensk-forfattningssamling/patientlag-2014821_sfs-2014-821). In addition, it is recommended that the patient and the next-of-kin are informed about the decision of refraining from CPR in the case of cardiac arrest (http://www.riksdagen.se/sv/dokument-lagar/dokument/svensk-forfattningssamling/patientlag-2014821_sfs-2014-821). According to a survey made in Sweden in June 2018 it was shown that there were significant shortages in the information regarding the DNACPR-decision given to patients and the next-of-kin (http://sverigesradio.se/sida/artikel.aspx?programid=83&artikel=6972774). This led to a national debate in the Swedish media about DNACPR-decisions.

In the Stockholm Region there are two Medical Home Care Units that are financed by the County Council; ASIH Stockholm Södra, hereafter referred to as “Unit A”, and ASIH Stockholm Norr, hereafter referred to as “Unit B”. The two Units are described in more detail in the methods section. At Unit A, written guidelines about decision taking and documentation regarding the DNACPR-decision were introduced in 2017. These guidelines included a requirement to take a decision about CPR (CPR: yes or no), which had to be stated in the medical records. Unit B had no such obligatory requirement about CPR-decisions.

The aim of this study was to describe the frequency of decision and documentation related to DNACPR in these two Medical Home Care Units in Stockholm and to investigate if the requirement for a CPR-decision influenced the frequency of DNACPR-decisions.

## Methods

### Description of the two medical home care units from this study

The two Medical Home Care Units in this study enrol the same kind of patients and have the same commission from the Swedish National Health Authorities. Unit A has approximately 380 enrolled patients and Unit B has approximately 280 enrolled patients on any given day. The median care time at the Units is approximately 3–4 months. In addition to the Medical Home Care facilities, both Units have an in-patient Hospice Ward for mainly end-of-life patients with short lifetime expectancy. The Hospice Ward at Unit A has 16 beds and Unit B has 12 beds. The median care time at the Hospice Wards are 10–14 days.

There are 20 senior consultants working at Unit A and 18 working at Unit B. The senior consultants have different backgrounds, but all have 5 years of specialist training in one of the disciplines: oncology, family medicine, internal medicine, geriatrics, clinical pharmacology, nephrology, hematology or anesthesia. Palliative Medicine is an additional specialty in Sweden (2 years training after the 5 years of specialist training), and approximately 50% of the senior consultants in both Units had specialist training in Palliative Medicine.

At Unit A it was mandatory to state in the medical record what should be done in the case of cardiac arrest (CPR: yes/no) according to new written guidelines introduced in 2017. Unit B had chosen not to have such a requirement for CPR-decision documentation. Both Units had the same electronic system for medical records and the same documentation templates. At both Units the decision about DNACPR (if present) was stated in a specific subheading in a template called “Individual Care Plan” that is present for all patients enrolled at both Units.

Both Units had joint education about the poor outcome of CPR in patients being in palliative phase of their disease. In addition, staff from Unit A had additional meetings discussing the new guidelines regarding the requirement to take a specific decision about CPR, how to document the decision in the medical records, and information that the decision should be regularly evaluated.

### Review of medical records

A retrospective study was performed to compare the frequency of decision taking and documentation of DNACPR in the two Medical Home Care Units. All medical records were reviewed at each Unit on a given day.

At Unit A, one physician (PL) reviewed all “Individual Care Plans” at a specific date in January 2019. At Unit B, one physician (BS) reviewed all “Individual Care Plans” for all patients at the Unit on a randomly selected day in November 2018. The review comprised both out-patients enrolled to the Advanced Medical Home Care and in-patients at the Hospice Wards.

The review of medical records was performed approximately 1.5 years after the introduction of written guidelines on decision making and documentation at Unit A.

For each patient the following data was collected: palliative phase of disease (yes/no), documentation of what to do in case of cardiac arrest and the decision of DNACPR (yes/no), documentation if the patients were informed about the decision (yes/no or unknown) and whether the next-of-kin was informed about the decision (yes/no or unknown).

### Definition of patients in palliative phase

Only patients being in palliative phase of their disease were included in the final analysis, i.e. patients suffering from a life-limiting disease with a short life-time expectancy and in need of palliative care. This includes patients suffering from an advanced cancer, but also patients with other life-threatening diseases with a short lifetime expectancy, i.e. patients with late-stage heart failure, late-stage chronic obstructive pulmonary disease (COPD) and late-stage kidney failure. In case of uncertainty as to whether the patients were in palliative phase or not, the medical records were reviewed by two different physicians (LBB and PL for patients in Unit A, and by LBB and BS for patients in Unit B) and a joint decision about whether a patient could be defined as being a palliative phase or not was taken.

### Statistical analysis

Statistical analysis was performed using Graph-Pad Prism version 6.0. The comparison between the two Units (yes or no decision) was performed by Fishers exact test. A significance of *p* ≤ 0.05 was considered as statistically significant. The comparison between the two Units regarding patient age was performed using student’s t-test.

### Ethical statement

The review of the medical records was approved by the Regional Ethical Committee in Stockholm, Dnr 2018/1798–31. Both studies were also approved by the Unit Director at each Unit. All data extracted from the medical records was anonymised before analysis to ensure that no individual patient could be identified in the data-set or the analysis.

## Results

**Fig. 1:**
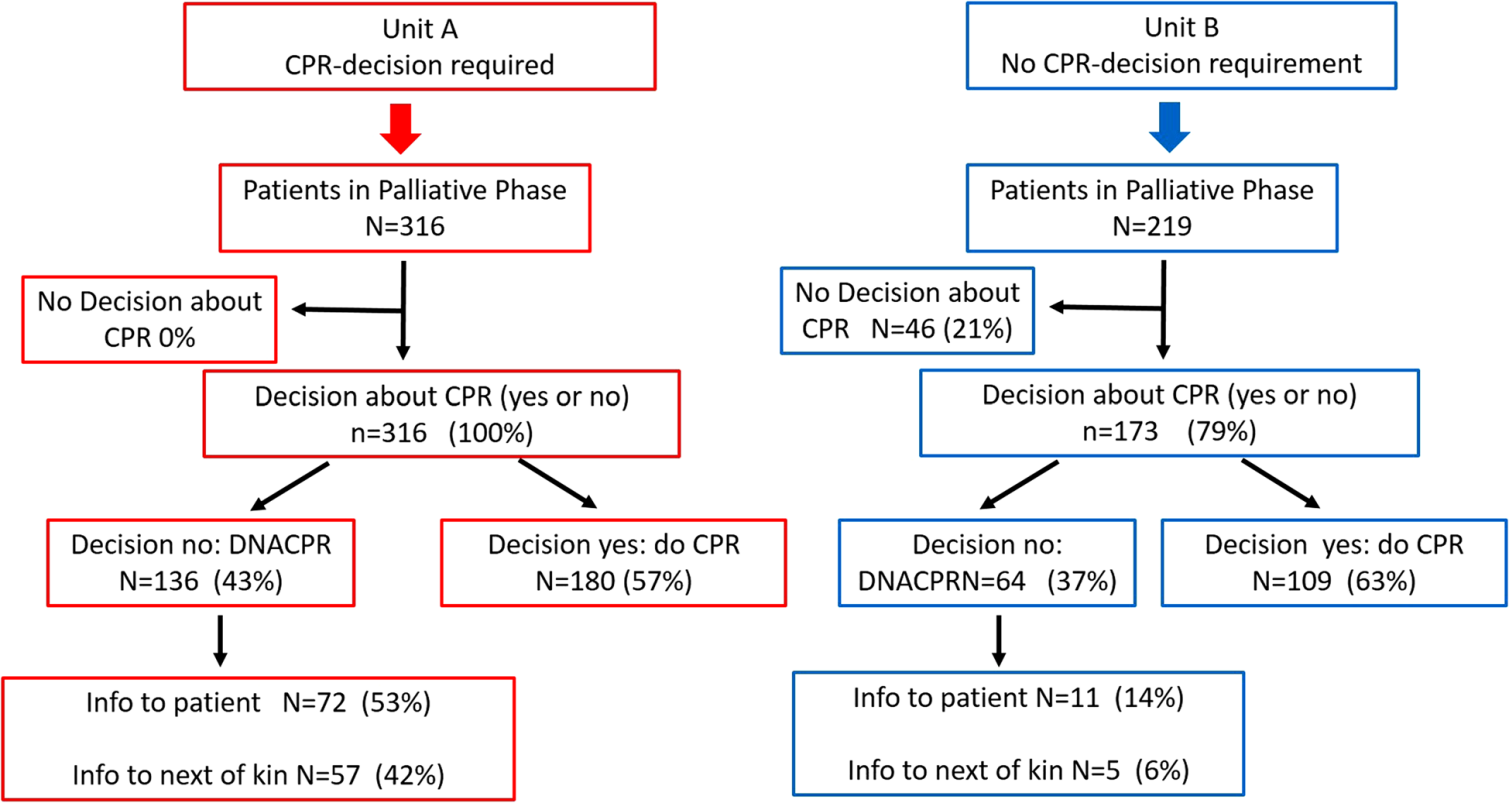
Flow chart of patients included in the study from the two Medical Home Care Units in Stockholm, Sweden, including the frequency of the presence of a decision and documentation about cardiopulmonary resuscitation (CPR) in the case of cardiac arrest

At Unit A, 372 patients were enrolled, of which 316 were in palliative phase. At Unit B, 272 patients were enrolled on the specific day, and 219 were in palliative phase of their disease (Fig. 1). Most of the patients in the Units were cancer patients. The demographic data of the study population is presented in Table 1.

**Table 1:** Demographic data of the patients in palliative phase, enrolled at two Medical Home Care Units in Stockholm, that were included in this study

	Unit A (CPR-decision mandatory)	Unit B (CPR-decision not mandatory)	*p*-value
Patients in palliative phase	*N* = 316	*N* = 219	NA
Average age (range)	69 years (23–95)	73 years (18–99)	*p* = 0.88
Men	49% *N* = 155	49% *N* = 108	*p* = 1.00
Women	51% *N* = 161	51% *N* = 111	*p* = 1.00
Decision about CPR (yes or no)	100% *N* = 316	79% *N* = 173	*p* < 0.001
Decision of “attempt CPR”	57% *N* = 180	50% *N* = 109	*p* = 0.11
Decision of “do not attempt CPR” (DNACPR)	43% *N* = 136	37% *N* = 81	*p* = 0.21
Decision of DNACPR In-patients / Hospice Ward	100% *N* = 16	100% *N* = 12	*p* = 1.00
DNACPR decision: Information to patient	53% *N* = 72	14% *N* = 11	*p* < 0.001
DNACPR decision: Information to next-of-kin	42% *N* = 57	6% *N* = 5	*p* < 0.001

Abbreviations: *CPR* Cardiopulmonary resuscitation, *DNACPR* Do not attempt CPR, *ns* not statistical significant. *NA* not applicable

**Fig. 2:**
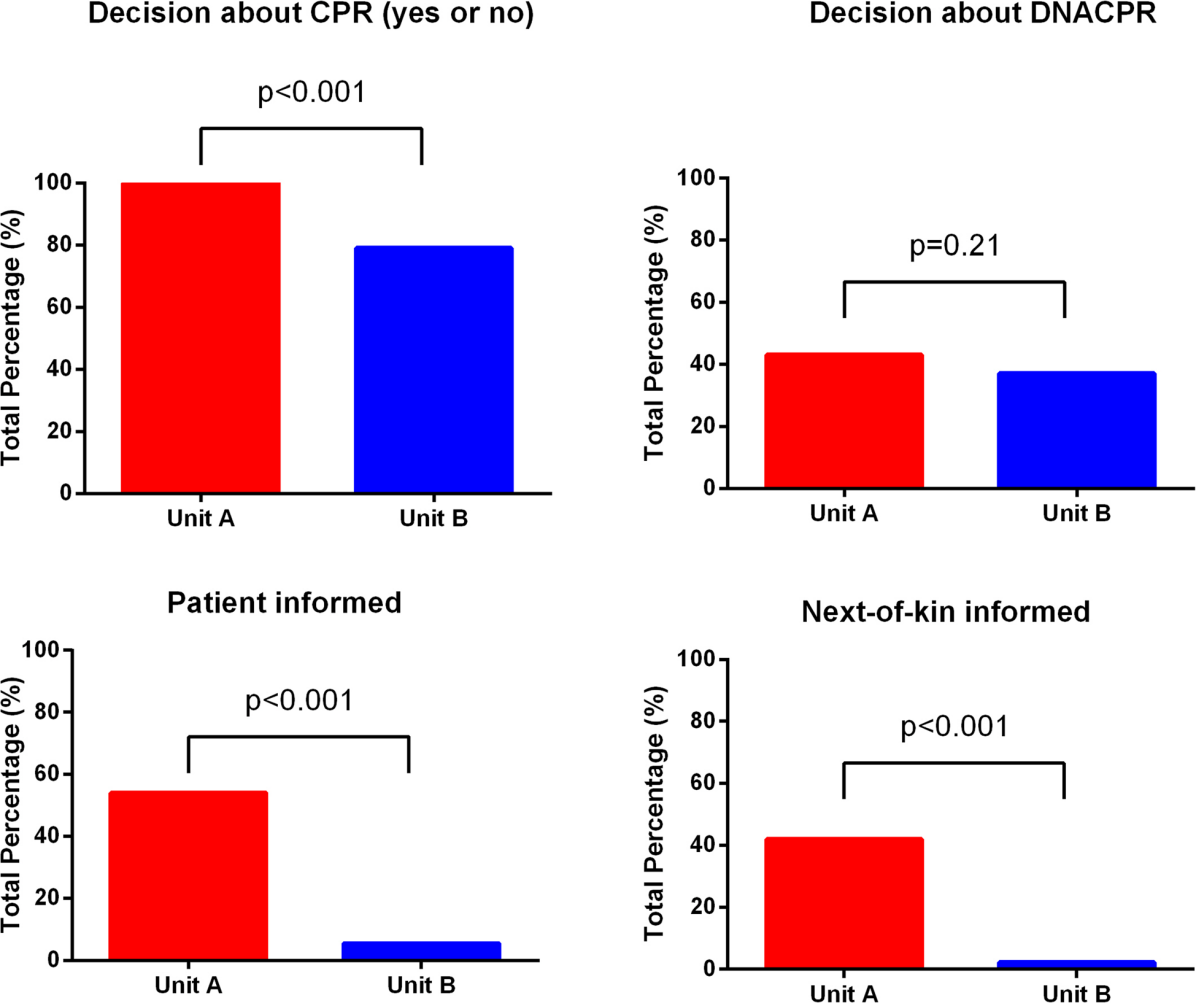
Graphical representation of the percentage of patients in palliative phase at two Medical Home Care Units in Stockholm, Sweden, that had documentation in their medical records about a decision regarding cardiopulmonary resuscitation (CPR) in the case of cardiac arrest, a decision about Do not attempt CPR (DNACPR) and documentation about if the patient or next-of-kin were informed about the DNACPR-decision. In Unit A, a CPR-decision was mandatory in the medical records according to written guidelines while no such requirement was present in Unit B. Statistical analysis was performed using Fischer’s exact test

Among all patients defined as being in palliative phase of their disease, 100% had a decision about CPR (yes/no) at Unit A and 79% at Unit B, as shown in Fig. 1. The difference between the two Units was statistically significant (*p* < 0.001) (Fig. 2).

At Unit A, 43% of the patients had a decision about DNACPR in the case of cardiac arrest and at Unit B there were 37% of patients who had taken this decision. This difference was not statistically significant (*p* = 0.88), (Fig. 2). An “attempt CPR-decision” was present in 57% of all patients in palliative phase at Unit A compared to 50% at Unit B, (*p* = 0.11) (Table 1).

At the in-patient Hospice Wards all patients had a DNACPR-decision in both Units, *n* = 16 and *n* = 12 respectively (Table 1).

In the cases where the decision about DNACPR had been taken, it was documented in the medical record that the patients had been informed in 54% of the cases at Unit A, but was only documented in the medical records of 14% of the patients at Unit B. Documentation about information to next-to-kin was present in 42% of cases at Unit A and 6% of cases at Unit B (Fig. 2).

## Discussion

This study showed that the decision of DNACPR in patients in palliative phase of their disease enrolled to two Medical Home Care Units in Stockholm is documented in less than 50% of cases. Written guidelines about DNACPR-documentation and the requirement for a CPR-decision did not increase the frequency of DNACPR decision taking.

It could be hypothesized that the presence of written guidelines would encourage the physicians to take more DNACPR-decisions, but this was not observed in this study. Instead, the physicians were more accurate to document what to do in the case of cardiac arrest, and all patients had a decision, i.e. “attempt CPR” or “do not attempt CPR” at Unit A. A total of 57% of all patients at Unit A being in palliative phase of the disease had an “attempt CPR-decision” compared to 50% at Unit B. In Unit B, 21% of the patients lacked documentation of formal decision about what to do in case of cardiac arrest and thus the physician would be required to take an on-the-spot decision in the case of cardiac arrest.

Moreover, the staff at Unit A had had meetings discussing the new guidelines and this did not results in a higher frequency of DNACPR-decision but improved the documentation.

As described in the introduction, the outcome when performing CPR to patients with life-limiting diagnosis is very poor and often unsuccessful [4, 9]. The success rate of CPR in the general Swedish population in the case of cardiac arrest has been reported to be approximately 10% [2, 6]. The success rate of CPR in patients with life-limiting illnesses has never been studied in a Swedish population but is likely very low, comparable with reports from other countries i.e. 0–2% [4, 9]. However, the decision not to perform CPR is difficult to take even if the physicians are well-informed about the poor outcome of CPR.

Studies on the decision of CPR (yes or no) is vastly different between countries. In a study performed on 151 in-hospital patients in New Zealand, 27% had a decision about CPR (yes/no) in the medical records [10]. In a Canadian study on 205 patients referred to palliative radiotherapy, a DNACPR-decision had only been taken in 4% of cases, and an “attempt CPR-decision” had been taken in 3% of cases, while in 93% of cases no decision was taken [11].

Our results are in contrast to a study performed in the UK showing a statistically significant increase in CPR decisions after the introduction of a Form of Treatment Options for patients where the CPR-decisions increased from 52 to 77% [12].

According to several studies, patients with terminal illnesses want to be involved in the decision of DNACPR and the care planning [13, 14]. Moreover, it has been shown that there is often a miss-match between the patient’s preferences for CPR and the physician’s perceptions about the wish of the patient [15]. The results from these studies emphasize the importance of physician-patient discussion about CPR. In the home care setting there is also a need for involving the next-of-kin in the discussion of CPR since they are often present at the time of death [1]. The present study does not answer the question about the involvement of the patient or of the next-of-kin in the decision taking but the results suggest that most patients and next-of-kin were not informed about the decision.

It should be noted that it is more common that non-oncological patients with terminal illnesses are exposed to CPR at time of death compared to oncological patients [16–19]. Thus, it is particularly important to identify non-oncological patients who are in the end-of-life stage, and to ensure that the physician has the courage to discuss the DNACPR-decision with them in order to avoid the suffering connected to the CPR-measures at time of death [20–22].

In Wales, UK, a new approach to tackle the sensitive issue of DNACPR discussions was developed in 2015 called “TalkCPR” [23]. This educational program included websites, videos and media pads aimed at both patients and healthcare professionals [23]. This approach was very successful, and physicians exposed to the education reported that they discussed DNACPR more frequently with their patients and felt more comfortable with the discussion.

Many patients have a poor knowledge of CPR and the majority overestimate the success rate of CPR [14, 24]. Notably, physicians also often overestimate the success rate of CPR [24]. In a study where in-patients were randomized to watch a video about CPR or about “standard care”, the patients that watched the video were less likely to want CPR in the case of cardiac arrest in comparison with the group exposed to “standard care” [25]. The study indicated that appropriate patient education might facilitate the decisions and the discussion about DNACPR.

### Limitations

This study has several limitations. First, the study is a retrospective, observational study on the frequency of documentation of DNACPR and no adjustments for possible cofounding factors were made. However, the two Units enroll patients with similar conditions and have the same commission from the Stockholm County Council. The physicians at both Units had been exposed to the same education about CPR. The only difference between the Units was the presence of written guidelines at the Unit A including the requirement for a CPR-decision to be taken. Finally, it should be stressed that although it was not documented in the medical records, both patients and next-of-kin may have been informed about the DNACPR-decision. Since there were no guidelines that urged the physician to write the decision in the medical records in Unit B this might influence the result of what was found in the medical records and may not necessary mean that no information had been provided. In Stockholm the patient has access to their own medical records online since 2018. Thus, the patients have the possibility to find the information about the CPR-decisions by themselves and as such the physicians may not think it was necessary to note in the medical records that the patient was informed.

### Recommendations and future perspectives

A study performed in Taiwan showed that the phrasing in the medical record “to allow natural death” versus “do not resuscitate” was perceived as positive and easier to accept when a decision about not attempting CPR was to be taken [26]. Perhaps this phrasing would make it easier for the physician to write the DNACPR-decision in the medical record and this could be a good option in Sweden where the patient has online access to their medical records.

The results presented here show that education of the physicians about poor outcome of CPR and written guidelines about decision making was not enough for more DNACPR-decisions to be taken. Previous studies show the importance of education of the patients about the outcome of CPR [13, 14, 23]. Thus, in future studies a prospective design should be employed with an intervention including different educational approaches for patients about CPR. In addition, a study exploring factors and views that influence the physician’s decision about DNACPR in Medical Home Care is warranted.

## Conclusion

This study showed that less than 50% of patients considered as being in palliative phase of their disease had a decision of DNACPR in two Medical Home Care Units in Stockholm. Written guidelines and the requirement for a CPR-decision did not increase the overall frequency of DNACPR decisions but were associated with a higher frequency of documentation in the medical records about decisions taken and information given to patients and the next-of-kin.

## Data Availability

The raw-data is available from the corresponding author upon request.

## Abbreviations

CPR: Cardiopulmonary resuscitation
DNACPR: Do not attempt CPR
